# Primary Amebic Meningoencephalitis Caused by *Naegleria fowleri*, Karachi, Pakistan

**DOI:** 10.3201/eid1702.100442

**Published:** 2011-02

**Authors:** Sadia Shakoor, Mohammad Asim Beg, Syed Faisal Mahmood, Rebecca Bandea, Rama Sriram, Fatima Noman, Farheen Ali, Govinda S. Visvesvara, Afia Zafar

**Affiliations:** Author ffiliations: Aga Khan University Hospital, Karachi, Pakistan (S. Shakoor, M.A. Beg, S.F. Mahmood, F. Ali, A. Zafar);; Centers for Disease Control and Prevention, Atlanta, Georgia, USA (R. Bandea, R. Sriram, G.S. Visvesvara);; Liaquat National Hospital, Karachi (F. Noman)

**Keywords:** Naegleria fowleri, Pakistan, ameba, encephalitis, parasites, primary amebic meningoencephalitis, water, contamination, dispatch

## Abstract

We report 13 cases of *Naegleria fowleri* primary amebic meningoencephalitis in persons in Karachi, Pakistan, who had no history of aquatic activities. Infection likely occurred through ablution with tap water. An increase in primary amebic meningoencephalitis cases may be attributed to rising temperatures, reduced levels of chlorine in potable water, or deteriorating water distribution systems.

Primary amebic meningoencephalitis (PAM) is a fatal disease caused by the thermotolerant free-living ameba *Naegleria fowleri*. Found worldwide in moist soil and freshwater, these amebae proliferate during summer when ambient temperature increases. The organism enters the nasal cavity when water contaminated with amebae is aspirated. Subsequently, it invades the central nervous system through the olfactory neuroepithelium and causes a fatal infection that clinically resembles acute bacterial meningitis. We report 13 cases of *N. fowleri* PAM in a period of 17 months in the coastal city of Karachi, Pakistan.

## The Study

In June 2008, a 30-year-old, previously healthy man was referred to the Aga Khan University Hospital with a 2-day history of high-grade fever, severe headache, and seizures. He was comatose with a fixed and dilated left eye pupil. Magnetic resonance imaging showed basal meningeal enhancement. A lumbar puncture found an opening pressure of 44 cm H_2_O. Cerebrospinal fluid (CSF) analysis showed low glucose (<5 mg/dL), high protein level (1,028 mg/dL), and lymphocytic pleocytosis (900 cells/mm^3^ with 85% lymphocytes). Gram stain and latex agglutination (LA) test results were negative for bacteria. Wet film of CSF showed motile amebic trophozoites, but CSF volume was insufficient for amebic culture. A preliminary diagnosis of PAM was made, and therapy was begun with intravenous amphotericin B plus oral rifampin and fluconazole. The patient died 4 days after admission.

In September 2008, a previously healthy 25-year-old man was admitted with a 24-hour history of fever, vomiting, and neck rigidity. CSF analysis indicated hypoglycorrhachia, elevated protein level, and neutrophilic pleocytosis. Gram stain and LA test results were negative for bacteria, and wet preparation of CSF showed motile amebic trophozoites. Ptosis of the left eye and a fixed dilated pupil developed on the day of admission, and he was intubated for airway protection. Despite therapy with intravenous amphotericin B, oral fluconazole, and rifampin, his condition deteriorated, and he died 14 days after admission. CSF was cultured on nonnutrient agar on a lawn of *Escherichia coli* American Type Culture Collection (Manassas, VA, USA) 29522 in Page ameba saline (*1*). Cultured amebae produced flagellates in distilled water at 37°C within 15–30 min.

On the basis of this apparent upsurge of cases, a laboratory policy was instituted at the Aga Khan University Hospital to perform wet mounts of all processed CSF samples that were consistent with bacterial meningitis but had negative Gram stain and LA test results. Although no new cases of PAM were detected in 2008, 11 were identified from April through November 2009. Case-patients were referred from tertiary-care hospitals in Karachi (Table).

**Table:** Clinical features and CSF analysis results for 13 patients with *Naegleria fowleri* meningoencephalitis, Karachi, Pakistan, 2008–2009*

Characteristic	Patient nos., in order of treatment													Descriptive statistics†
	1	2	3	4	5	6	7	8	9	10	11	12	13	
Age, y	31	25	30	36	60	30	64	18	22	16	18	18	35	Mean 31.0 ± 15.33
Date treatment sought	2008 Jul	2008 Sep	2009 Apr	2009 May	2009 Jul	2009 Jul	2009 Jul	2009 Jul	2009 Aug	2009 Aug	2009 Oct	2009 Oct	2009 Nov	
Duration of illness before seeking treatment, d	2	1	2	4	4	1	2	5	2	2	2	3	3	Mean ± SD 2.5 ± 1.19
Duration of survival after symptom onset, d	6	15	3	6	7	9	7	6	5	3	3	7	6	Mean ± SD 6.38 ± 3.15
Fever	+	+	+	+	+	+	+	+	+	+	+	+	+	Present in 13/13 (100%)
Headache	+	+	+	+	+	+	+	–	+	+	+	+	+	Present in 12/13 (92.3%)
Seizures	+	–	+	–	+	–	+	–	+	+	+	+	–	Present in 8/13 (61.5%)
CSF analysis														
Glucose, mg/dL†	<5	42	2	<5†	26	<5†	<5‡	30	<5‡	<5‡	80	50	<5‡	Undetectable in 53.8%
Protein, mg/dL	1,028	418	909	774	504	1,147	1342	320	998	330	371	179	1,296	Mean ± SD 739.69 ± 405.29
Leukocytes, cells/mm^3^	900	900	5,976	2,000	2,500	7,500	5,200	6,000	840	6,500	150	185	11,750	Mean ± SD 3,877.0 ± 3,565.37
Neutrophils, %	15	90	90	90	60	80	90	90	90	96	75	25	95	Neutrophilic pleocytosis, 84.6
Lymphocytes, %	85	10	10	10	40	20	10	10	10	4	25	75	5	Lymphocytic pleocytosis, 15.4
CSF amebic culture	NA	+	+	+	+	+	+	+	+	+	+	+	+	NA
CSF PCR for *N. fowleri*	NA	NA	NA	NA	+	+	+	NA	NA	NA	NA	NA	NA	NA

*All patients were male except patient no. 11, who was female. CSF, cerebrospinal fluid; +, present; NA, no data available. †Statistics derived in SPSS software version 16.0 (LEAD Technologies, Charlotte, NC, USA). ‡Glucose <5 mg/dL= undetectable.

**Figure A1:**
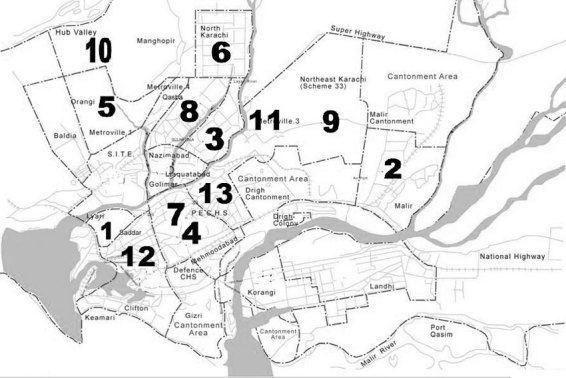
Map of Karachi, Pakistan, showing districts where patients 1–13 resided. Dotted-dashed lines indicate town boundaries; gray shading indicates drainage channels.

Twelve of 13 patients were male; ages ranged from 16 to 64 years (mean ± SD 31.0 ± 15.33 years). All were residents of Karachi but lived in different districts (Figure A1). Only 1 patient acknowledged a history of swimming. All patients’ conditions were treated with amphotericin B (1.5 mg/kg/d) and rifampin (600 mg/d). Most patients also received either fluconazole or itraconazole. All required intubation and ventilation within 24 hours of hospital admission, and treatment for PAM was started within 48–72 hours of admission. Nonetheless, only 2 patients survived for >5 days after admission; the mean ± SD time from symptom onset to death was 6.38 ± 3.15 days (range 3–15 days).

CSF samples from all patients (except the first) were positive for amebic culture. Furthermore, CSF samples collected from 3 patients in July 2009 were confirmed by real-time PCR for *N. fowleri* DNA at the Division of Parasitic Diseases, Centers for Disease Control and Prevention, Atlanta, Georgia, USA. Briefly, primers NaeglF192 (3′-GTG CTG AAA CCT AGC TAT TGT AAC TCA GT-5′) and NaeglR344 (5′-CAC TAG AAA AAG CAA ACC TGA AAG G-3′) were used to amplify a 153-bp fragment, detected by the hexachlorofluorescein (HEX)–labeled probe NfowlP (5′-HEX-AT AGC AAT ATA TTC AGG GGA GCT GGG C-BHQ1-3′). PCR was performed in a Mx3000P real-time thermocycler (Stratagene, La Jolla, CA, USA), with 2 initial incubations at 50°C for 2 min (incubation for uracil-DNA-glycosylase activity) and 95°C for 2 min (activation of Platinum *Taq* DNA-polymerase), respectively, followed by 40 cycles of 95°C for 15 s and 63°C for 60 s. Fluorescence was measured after each 63°C incubation. Results were analyzed by using Mx3000P version 2.0 software (*2*).

Domestic tap water was obtained for amebic culture from 2 patients’ homes (second and seventh patients in the series). Water samples (100 mL) were passed through a Millipore filter (Millipore Corp., Billerica, MA, USA), which was then inoculated face down on a nonnutrient agar plate with a lawn of *E. coli* as described above. Amebae were isolated from both water samples. However, only cultured amebae from the seventh patient’s sample were analyzed by real-time PCR, and *N. fowleri* DNA was detected in the sample.

## Conclusions

We report an increase of *N. fowleri* PAM cases in Karachi, Pakistan. Most cases were in healthy young adults with acute, fatal meningitis. Although enhanced case detection after instituting measures to improve the diagnosis of PAM may have contributed toward the rise in cases, sporadic previous reports from this region indicate that this is not the sole reason (*3**,**4*).

PAM is associated with freshwater swimming (*5*), and outbreaks have also been associated with poorly chlorinated swimming pools (*6*). However, all but 1 of the patients in this case series denied recent freshwater swimming or recreational water activities. Nevertheless, because all patients were Muslim, they routinely performed ritual ablution, which involves taking water into the nostrils. Infection acquired through this route has been reported (*7*). Because patients used domestic tap water for ablutions, we tested water from 2 patient’s homes and found *N. fowleri* amebae by culture. Additionally, *N. fowleri* DNA was identified by real-time PCR in a water sample from 1 patient’s home. PAM, resulting from aspiration of untreated ground water containing *N. fowleri* amebae, has been reported in 2 children from Arizona (*8*).

The presence of *N. fowleri* DNA in the ground water supply has been described (*9*). *N. fowleri* infections have also been described in children from Australia after exposure to water transported through overland pipes (*10*). The presence of *N. fowleri* amebae n Karachi’s municipal water supply may have several explanations. Karachi lacks an indigenous water source, and water obtained from 2 suburban freshwater lakes is not adequately filtered or chlorinated (*11*). Moreover, frequent leaks in water and sewage pipes can cause seepage of sewage into the water supply (*12*), which may be a potential reservoir for *N. fowleri* amebae (*13*).

Demonstrating *N. fowleri* amebae in the water supply, however, does not explain the sudden increase in the number of cases. *N. fowleri* amebae are thermotolerant, and outbreaks have been linked to steep rises in temperature (*14*). Recent temperature records from Karachi have also shown a temperature surge, and a further rise is expected in future summers (*15*). Our patients were also exposed to higher average monthly temperatures (Figure 1). Several factors may have precipated this outbreak: an increase in the number of pathogens (due to rising temperatures), changes in the ecosystem of the lakes, and further deterioration in the quality of water treatment and distribution systems.

This report highlights the emergence of fatal *N. fowleri* infection in a megacity. Changing climatic conditions may have contributed toward this sudden upsurge, which has serious consequences for the public at large. Urgent epidemiologic investigation into relevant environmental factors is needed to identify reasons for this sudden rise in PAM cases.

## Figures and Tables

**Figure:**
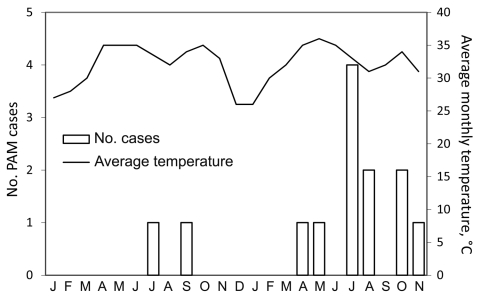
Primary amebic meningoencephalitis (PAM) cases seen in 2008 and 2009 by month (white bars) and average monthly peak temperatures (black line), Karachi, Pakistan. Karachi monthly temperatures obtained from http://www.wunderground.com/global/PK.html.
